# Relevance of Recent Thymic Emigrants Following Allogeneic Hematopoietic Cell Transplantation for Pediatric Patients with Inborn Errors of Immunity

**DOI:** 10.1016/j.jtct.2025.02.003

**Published:** 2025-02-07

**Authors:** Daniel Drozdov, Xianghua Luo, Rebecca A. Marsh, Roshini S. Abraham, Christen L. Ebens

**Affiliations:** 1 Division of Blood and Marrow Transplantation, Department of Pediatrics, University of Minnesota, Minneapolis, Minnesota; 2 Division of Bone Marrow Transplantation and Immune Deficiency, Cincinnati Children’s Hospital Medical Center and University of Cincinnati College of Medicine, Cincinnati, Ohio; 3 Division of Hematology/Oncology, Children’s Hospital, Kantonsspital Aarau, Aarau, Switzerland; 4 Division of Stem Cell Transplantation, University Children’s Hospital Zurich, Zurich, Switzerland; 5 Biostatistics Core at Masonic Cancer Center, University of Minnesota, Minneapolis, Minnesota; 6 Division of Biostatistics and Health Data Science, School of Public Health, University of Minnesota, Minneapolis, Minnesota; 7 Pharming Healthcare, Warren, New Jersey; 8 Department of Pathology and Laboratory Medicine, Nationwide Children’s Hospital, Columbus, Ohio

**Keywords:** Immune reconstitution, Recent thymic emigrants, Reduced intensity, conditioning, Neothymopoiesis

## Abstract

**Background:**

Allogeneic hematopoietic cell transplantation (HCT) can be curative for many inborn errors of immunity (IEI). Timely neothymopoiesis is paramount to favorable clinical outcomes after HCT. Neothymopoiesis can be quantified by flow cytometric measurement of circulating recent thymic emigrants (RTE; CD31+CD4+CD45RA+ T cells).

**Objectives:**

We hypothesized that decreased RTE would be associated with baseline HCT characteristics of older age at time of HCT and exposure to greater HCT conditioning intensity, as well as with HCT outcomes including mixed (<95%) lymphoid donor chimerism and presence of acute graft-versus-host disease (GvHD).

**Study Design:**

In this retrospective analysis two cohorts of pediatric IEI HCT recipients were identified at two centers that collected RTE data following allogeneic HCT. For both cohorts, patient and HCT information was recorded including but not limited to patient age, lymphoid donor chimerism, and occurrence of acute GvHD. Mixed effects models were fitted for the repeated measures of RTE with these covariates and time.

**Results:**

Between 2012 and 2021, a total of 162 pediatric IEI HCT recipients transplanted across both cohorts were eligible for inclusion. Cohort A (n = 34) included 23 males (68%). Median age at HCT was 2.2 years (interquartile range (IQR) 0.8 to 10.8). Eight (23.5%) underwent reduced intensity (RIC), 23 (67.7%) reduced toxicity myeloablative (RTC), and 3 (8.8%) myeloablative (MAC) conditioning. All received alemtuzumab serotherapy. Cohort B (n = 128) included 87 males (68%). Median age at HCT was 1.4 years (IQR 0.7 to 5.3). Seventy-six (59.4%) underwent RIC, 38 (29.7%) RTC, and 14 (10.9%) MAC. RIC and RTC patients received alemtuzumab serotherapy, MAC antithymocyte globulin. In the linear mixed effect model for RTE at 1 year after HCT for Cohort A, significant negative associations included increasing age (*P* < .0001) and RTC compared to RIC (*P* < .01). In the linear mixed effects model for RTE at 1 year after HCT for Cohort B, significant negative associations included increasing age (*P* < .0001), grade 2 to 4 acute GvHD (compared to grade 0 to 1; *P* < .01), MAC compared to RIC (*P* < .0001), MAC compared to RTC (*P* < .01), and RTC compared to RIC (*P* = .03).

**Conclusions:**

Serial measurement of RTE is a useful assessment of thymic function after HCT. In pediatric patients with IEI, older age at transplantation, greater intensity of conditioning, and occurrence of grade 2 to 4 acute GvHD were strongly associated with slower thymic-derived immune reconstitution. Mixed lymphoid donor chimerism was not associated with RTE in the linear mixed effects model. In addition to augmenting current anticipatory guidance on HCT outcomes, these findings may guide personalization of regimens to optimize clinical outcomes in IEI HCT.

## BACKGROUND

Allogeneic hematopoietic cell transplantation (HCT) provides a curative option for a wide array of inborn errors of immunity (IEI), with successful outcomes relying on the reconstitution of the immune system [1–3]. However, a significant challenge to successful immune reconstitution is the impairment of thymic function recovery. The ability of the thymus to regenerate naïve T cells from donor hematopoietic stem cells is crucial for ensuring long-term immunity with a recipient self-tolerant, diverse peripheral T cell repertoire [4–6]. Recent thymic emigrants (RTEs), characterized by surface expression of CD31+CD4+CD45RA+, are a critical marker of thymic output, providing a window into the recovery of thymic function after HCT. In response to signals from lymphodepleted niches following HCT, these RTE undergo homeostatic proliferation, during which CD31 is cleaved. Therefore, RTE, with a high content of T-cell receptor excision circles, represent a transient, truly naive centrally derived T cell population [7].

A lack of adequate thymic recovery after HCT can result in poor clinical outcomes, including increased susceptibility to infections, graft-versus-host disease (GvHD), and treatment-related mortality [6,8–10]. Recent studies have confirmed the importance of RTEs as biomarker of thymic output and measure of overall immune health post-HCT [9–13].

Many factors relevant to HCT are known to impact thymic output. Pediatric patients have a greater capacity for thymic recovery compared to adults, primarily due to the relative preservation of thymic function [14–16]. Neothymopoiesis can also be negatively impacted by thymic damage from HCT conditioning agents. While reduced intensity-conditioning (RIC) and reduced toxicity myeloablative conditioning (RTC) regimens aim to minimize organ toxicity and preserve thymic function, evidence regarding benefit to neothymopoiesis remains limited [17–21]. Understanding how these regimens affect thymic function is critical in optimizing post-HCT outcomes.

Thymic recovery is further hampered by the occurrence of GvHD. Acute GvHD is particularly damaging to the thymic epithelium, reducing thymic output [22,23]. Secondarily, treatment of GvHD with immunosuppression further delays immune reconstitution. Chronic GvHD has been also been shown to impair production of functional naïve T cells [22]. Consequently, efforts to reduce GvHD through improved immunosuppression regimens (e.g. inclusion of abatacept or posttransplant cyclophosphamide) or graft manipulation (T-cell or TCR*α*/*β* depletion) continue to be a major focus in the field of HCT [24–26]. Further research is required to elucidate the mechanisms by which GvHD and its treatment affect thymic function and how this can be mitigated to improve patient outcomes [27–29].

Moreover, the emergence of RTE seems to mark a decisive point in the evolution of long-term chimerism. Patients with residual host T-cell chimerism who rapidly acquire full donor lymphoid chimerism with the emergence of RTE, demonstrate complete long-term donor chimerism [30]. On the other hand, if the emergence of RTE in peripheral blood is not associated with a switch to full donor chimerism in the lymphoid compartment, loss of full donor chimerism in the myeloid compartment will ensue, generally with a lag phase of 3 to 6 months [31]. Consequently, chimerism analysis in RTE may be a predictor of long-term engraftment [32,33].

This study reports the methodology of circulating RTE quantification from two laboratories. We further explore the dynamics of RTE recovery in pediatric IEI HCT and associations with the complex factors known to influence thymic reconstitution, including patient age, conditioning regimen intensity, and the occurrence of GvHD. We additionally investigate association of RTE with achievement of full donor lymphoid chimerism. By analyzing these factors, we seek to enhance our understanding of immune reconstitution, particularly in the context of thymic regeneration. Such insights could lead to optimization of HCT timing, conditioning protocols, GvHD prophylaxis to promote faster and more complete immune recovery and reduced immune-mediated HCT complications for pediatric patients with IEI.

## STUDY DESIGN/ METHODS

### RTE Measurements

We collected RTE data prospectively in all patients. The RTE measurements were performed at day +100, day +180 and 1-year post transplant.

### RTE Assay Development

Pediatric normal values and percentiles for a flow cytometry based RTE assays were developed in 2 laboratories, Mayo Clinic Laboratories (Rochester, MN; Laboratory 1) and the Diagnostic Immunology Laboratory at Cincinnati Children’s Hospital Medical center (Cincinnati, OH; Laboratory 2). At Laboratory 1, whole blood was collected in etheylenediaminetraacetic acid (EDTA) tubes. Blood samples underwent red blood cell lysis, and were then incubated with a fluorophore-conjugated antibody cocktail for surface identification of CD45, CD3, CD4, CD45RA, CD45RO and CD31 (Becton Dickinson, San Jose, CA). Cells were fixed with 1% paraformaldehyde and run on a BD FACS Canto II^®^ for acquisition and analyzed with FACS Diva^®^ software (BD). Patient and healthy control samples were analyzed similarly. The absolute counts were obtained using BD TruCount^®^ tubes with CD45 and CD4. At Laboratory 2, whole blood was collected in EDTA tubes. Whole blood samples were incubated with a fluorophore-conjugated antibody cocktail for surface identification of CD45, CD3, CD4, CD8, CD45RA, CD45RO, CD27, CCR7, CD57, and CD31 (all antibodies purchased from BD, San Jose, CA or BioLegend, San Diego, CA). Cells were fixed and lysed with FACS Lysing Solution (BD, San Jose, CA) and run on a BD FACS Canto II^®^ or FACSLyric^®^ for acquisition and analyzed with FCS Express Flow Cytometry Software (De Novo Software). RTE were defined as CD45+, CD3+, CD4+, CD45RA+, CD27+, CD45RO−, CD31+ lymphocytes. The absolute counts were derived from concurrent absolute CD4+ T cell numbers from lymphocyte subsets.

### Patient Population

Pediatric (age 0 to 25 years at HCT) patients who underwent HCT treatment of an IEI between January 2012 and December 2021 at Cohort A (University of Minnesota M Health Fairview Masonic Children’s Hospital, Minneapolis, MN) and Cohort B (Cincinnati Children’s Hospital Medical Center, Cincinnati, OH) were assessed for inclusion in this retrospective analysis. From initial cohorts, patients were excluded if they received no or a nonstandard conditioning regimen, died before day +100 after HCT, and/or had no RTE data collected. Importantly, Cohort A utilized Laboratory 1 for RTE measurement; Cohort B utilized Laboratory 2, precluding combination of the cohorts.

### Ethics Approval

Institutional review boards of the University of Minnesota and Cincinnati Children’s Hospital Medical Center approved the study protocol and granted waiver of consent.

### Conditioning Regimen

A broad range of conditioning regimen intensities were used in this heterogenous patient population.

For Cohort A, reduced intensity conditioning (RIC) included fludarabine (30 mg/m^2^ IV per day for 5 days) and melphalan (140 mg/m^2^); reduced toxicity myeloablative conditioning (RTC) included fludarabine (40 mg/m^2^ IV for 4 days) and busulfan (IV every 6 hours for 4 days with a cumulative area under the curve (cAUC) pharmacokinetic goal of 72 mg × h/L); myeloablative conditioning (MAC) included busulfan (IV every 6 hours for 4 days with a cAUC goal of 72 mg × h/L) and cyclophosphamide (50 mg/kg IV per days for 4 days). In all regimens alemtuzumab was added as serotherapy on an intermediate dosing schedule [34] (RIC 0.2 mg/kg/day for 5 days from day −14, RTC and MAC 0.3 mg/kg for 3 days from day −12).

For Cohort B, RIC included fludarabine (30 mg/m^2^ IV per day for 5 days) and melphalan (140 mg/m^2^); RTC included the RIC regimen with addition of thiotepa (200 mg/m^2^), or fludarabine (30 mg/m^2^/day for 6 days) and busulfan (once daily IV for 4 days, with a cAUC goal of 55 to 65 mg × h/L); MAC included myeloablative dosing of busulfan (absolute dose of 16 mg/kg divided over 4 days without therapeutic drug monitoring) and cyclophosphamide (50 mg/kg IV per days for 4 days), with a single patient also receiving fludarabine. Serotherapy in Cohort B varied with intensity of conditioning, with subcutaneous alemtuzumab often provided on an intermediate dosing schedule with RIC and RTC, antithymocyte globulin with MAC.

### GvHD Prophylaxis

For GvHD prophylaxis, Cohort A used a combination of mycophenolate mofetil (day −3 to day +30) and a calcineurin inhibitor (day −3 to day +180, followed by a taper). Cohort B used a larger variety of strategies including this combination, other immunosuppressive agents, and/or T cell depletion (summarized in Table 1).

### HCT Variables

Baseline demographic and allogeneic HCT information including sex, IEI diagnosis, age at HCT, conditioning regimen, donor and recipient HLA match, stem cell source, and GvHD prophylaxis regimen were recorded. HCT outcomes including neutrophil recovery (absolute neutrophil count >500 cell/*μ*L × 3 consecutive days after nadir), and occurrence and severity of acute GvHD were recorded. Lymphoid donor chimerism by small tandem repeats or XY fluorescence in situ hybridization (FISH) was measured at 100 days, 6 months and 1 year after HCT.

### Statistical Analysis

Boxplots of RTE by time (100 day, 6 months, or 1 year) were presented for the variables of age (<1 year, 1 to 7 years, or ≥7 years old), conditioning regimen (MAC, RTC, or RIC), day +100 acute GvHD (grade 0 to 1 or grade 2 to 4) and day +100 lymphoid donor chimerism (dichotomized into full ≥95% or mixed <95%), and Wilcoxon rank sum test was used to test the difference in RTE across the different levels of these variables. A linear mixed model was performed for the repeated measures of RTE (after square root transformation) with covariates time, age at transplant (in years), conditioning regimen, day +100 acute GvHD, and day +100 lymphoid donor chimerism and all the two-way interactions with time. All analyses were performed in SAS 9.4 (SAS Institute Inc., Cary, NC). All tests were two-sided, and *P* values < .05 were considered statistically significant.

## RESULTS

**Figure 1.**
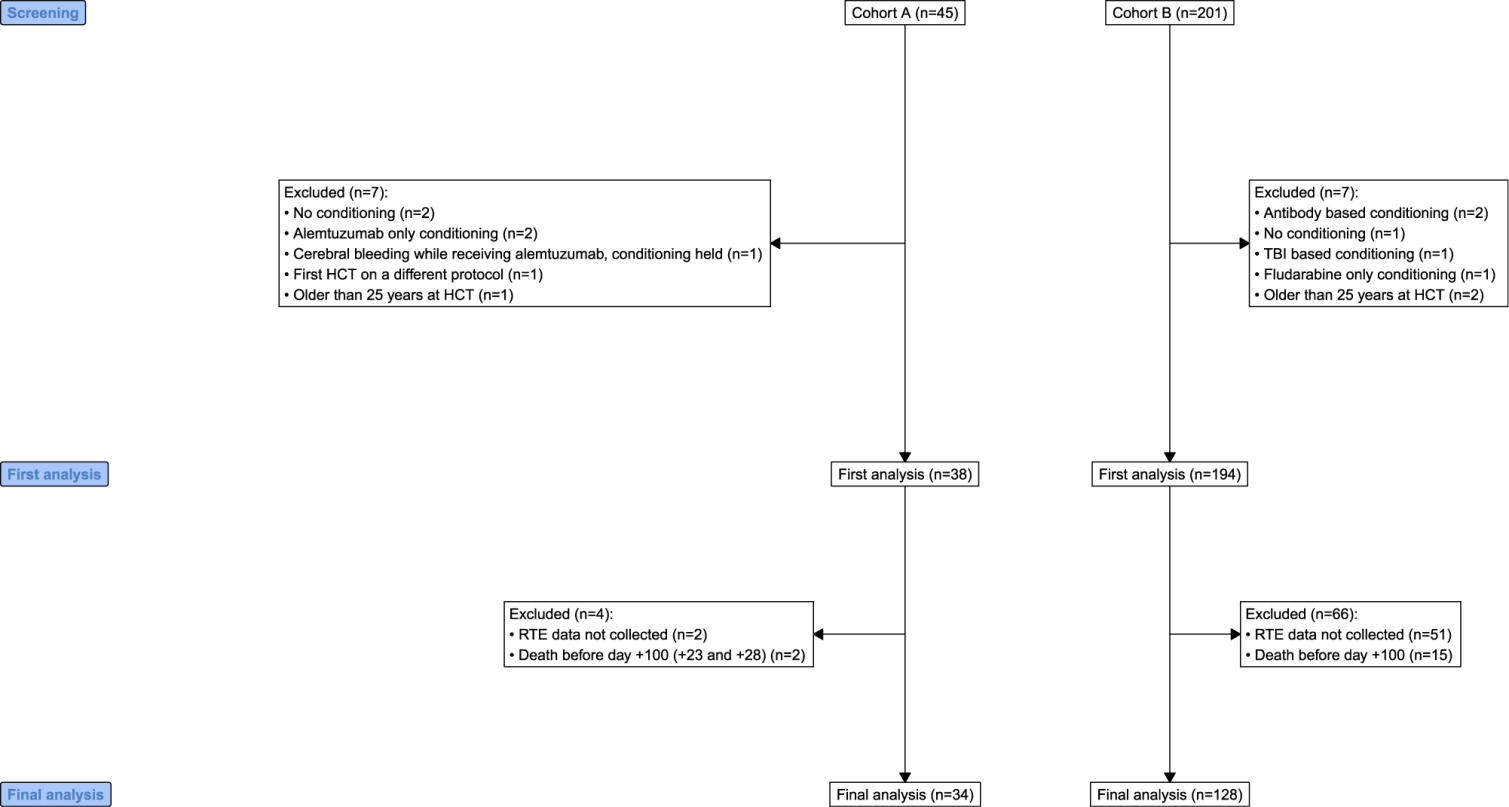
Consort diagram.

Between 2012 and 2021, forty-five patients with IEI were transplanted at Cohort A, and 201 at Cohort B. Eleven patients at Cohort A and 71 at Cohort B were excluded from the analysis as they did not meet prespecified inclusion criteria (Figure 1—*Consort Diagram*).

Cohorts A and B were similar in baseline characteristics with some variability in IEI diagnoses represented (detailed in Table 1). Cohort A (n = 34) included 23 males (68%). Median age at HCT was 2.2 years (IQR 0.8 to 10.8). Cohort B (n = 128) included 87 males (68%). Median age at HCT was 1.4 years (IQR 0.7 to 5.3). IEI diagnoses included chronic granulomatous disease, severe congenital neutropenia, CD40 ligand deficiency, severe combined immunodeficiency, Wiskott–Aldrich syndrome, hemophagocytic lymphohistiocytosis and other IEI. Distribution of conditioning intensities at sites further reflects these varied IEI HCT indications. At Cohort A, RIC, RTC, and MAC were used for 23.5%, 67.7%, and 8.8%, respectively. At Cohort B, RIC, RTC and MAC were used for 59.4%, 29.7% and 10.9%, respectively.

For recipients of RIC (n = 38) and RTC (n = 76) in Cohort B, subcutaneous alemtuzumab was provided for 97%, with 3 receiving no serotherapy. For 88% receiving RIC conditioning (67 of 76) and 78% receiving RTC fludarabine + melphalan + thiotepa conditioning (7 of 9), total alemtuzumab dose of 1 mg/kg was provided, typically dosed at 0.2 mg/kg/day over 5 days from day −14 (with the initial daily dose capped at 3 mg for those over 15 kg and the remainer of 1 mg/kg divided over the subsequent days). For RTC fludarabine + busulfan conditioning, twenty-seven of 29 (93%) received a total alemtuzumab dose of 0.5 mg/kg (dosed over 3 days from day −8). For Cohort B MAC (n = 14), horse ATG 15 mg/kg/day was used over 4 days from day −2 (day −2 to +1; total 60 mg/kg) in 10 (71%), rabbit ATG 2.5 mg/kg/day over 4 days from days −5 (total 10 mg/kg) in 2 (14%) patients, and no serotherapy in 1 (7%).

**Figure 2.**
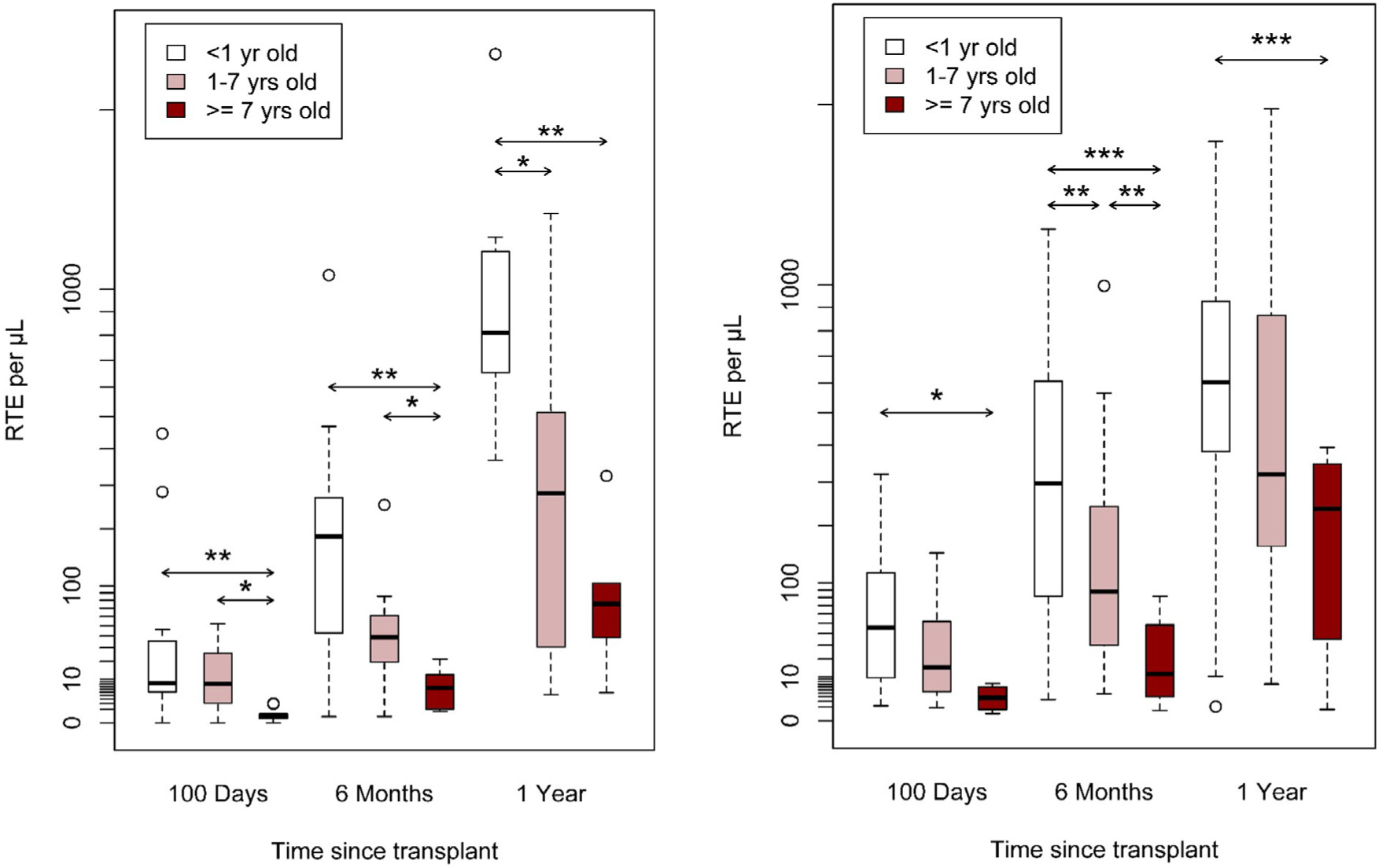
Cohort A (A) and Cohort B (B) absolute RTE by age group, *P < .05, **P < .01, ***P < .001.

**Figure 3.**
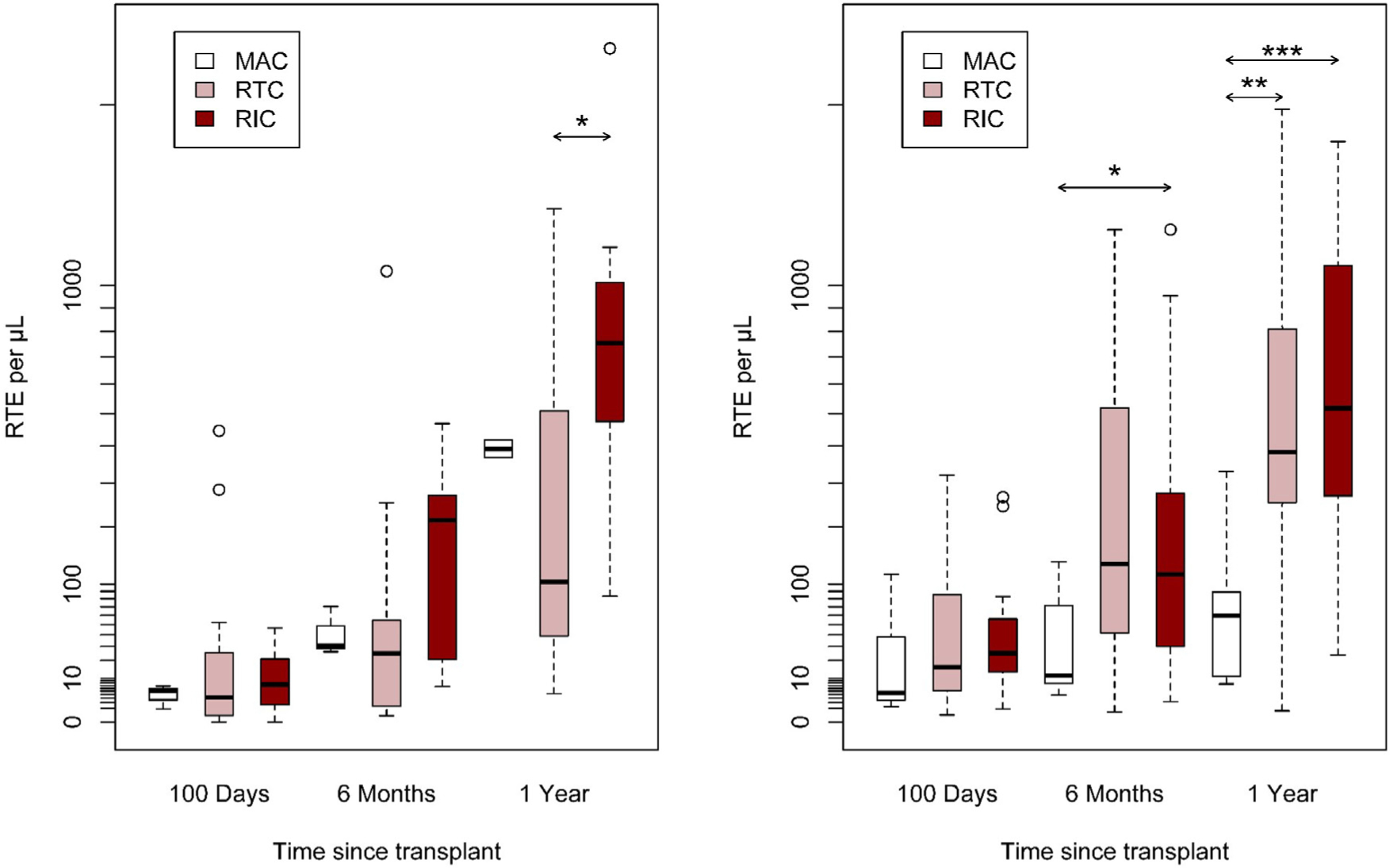
Cohort A (A) and Cohort B (B) absolute RTE by conditioning intensity over time, *P < 05, **P < .01, ***P < .001.

**Figure 4.**
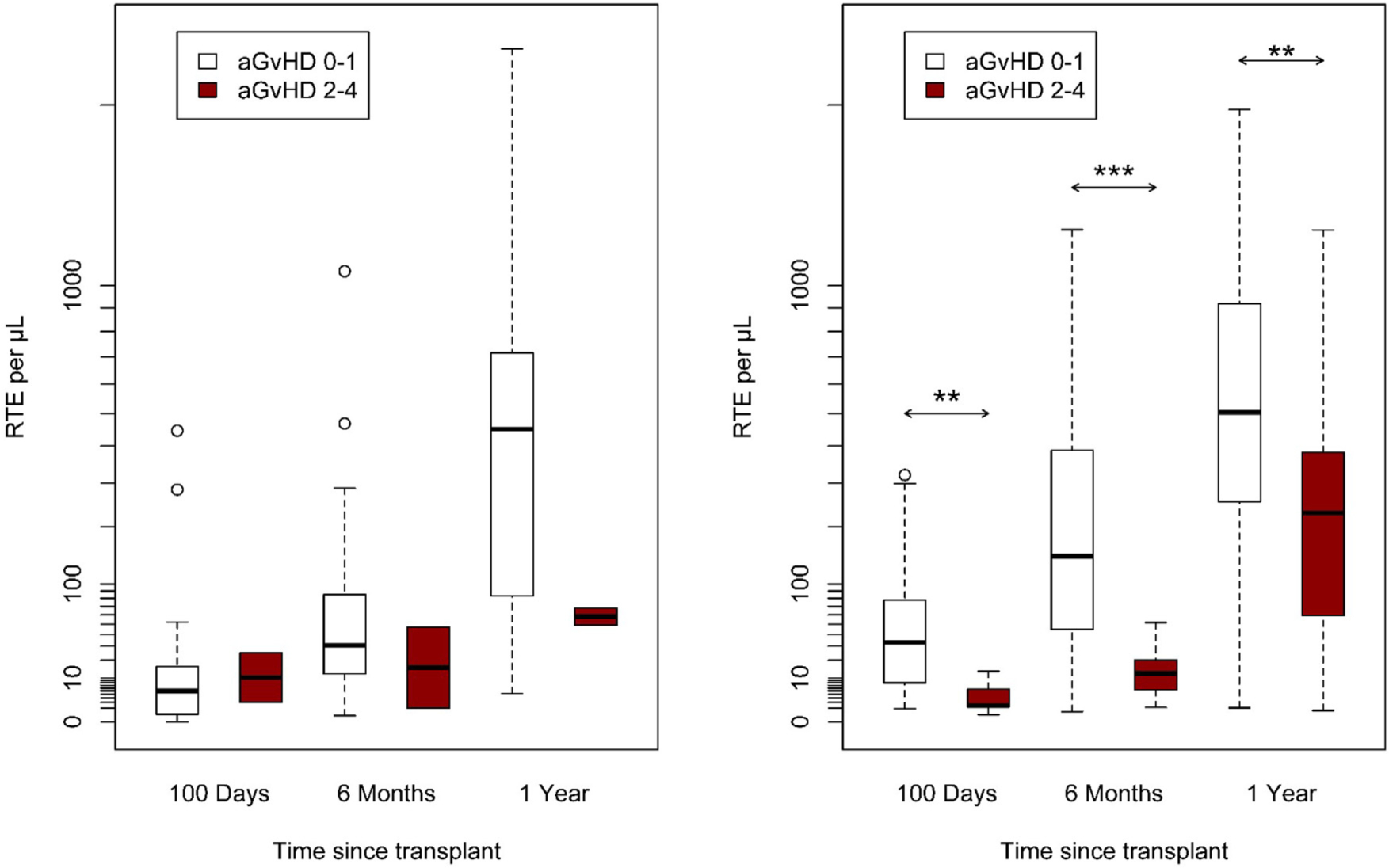
Cohort A (A) and Cohort B (B) absolute RTE by acute GvHD grade over time, *P < .05, **P < .01, ***P < .001.

Older patients at HCT had lower RTE absolute values at 1-year after HCT in both cohorts (Figure 2). Patients with more intensive conditioning had lower RTE absolute values at 1-year post-HCT than patients with less intensive conditioning regimen (Figure 3). Patients experiencing grade 2–4 acute GvHD at day +100 after HCT had lower RTE absolute values at 1-year after HCT in both cohorts (Figure 4). Wilcoxon rank-sum pairwise comparison statistics of univariate analyses are available in Supplementary Material.

In the linear mixed effect model for RTE at 1 year after HCT for Cohort A, significant negative associations included increasing age (*P* < .0001) and RTC compared to RIC (*P* < .01), while acute GvHD and lymphoid donor chimerism at day +100 were not significant (*P* > .05) (Table 2).

In the linear mixed effects model for RTE at 1 year after HCT for Cohort B, significant negative associations included increasing age (*P* < .0001), grade 2 to 4 acute GvHD (compared to grade 0 to 1; *P* < .01), MAC compared to RIC (*P* < .0001), MAC compared to RTC (*P* < .01), and RTC compared to RIC (*P* = .03). The linear mixed model with additional covariates of lymphoid donor chimerism at day +100 (and its interaction with time) found no significant difference between mixed and full chimerism group at any time point (*P* > .05) (Table 3).

## DISCUSSION

The reconstitution of a thymically educated diverse T cell repertoire after HCT is a critical determinant of clinical outcomes [6,11], particular in IEI populations. RTEs provide a readily accessible circulating measure of these newly produced T cells, reflecting thymic activity and a post-HCT reconstitution of one critical member of cellular immunity. The dynamics of RTE reconstitution are influenced by multiple factors, including patient age, conditioning regimen intensity, graft type, use of lymphocyte targeting serotherapy and the underlying disease and GvHD [13,27–29].

Age at transplantation is a critical factor influencing RTE reconstitution. Younger patients generally exhibit a more robust and rapid recovery of thymic function. In this study, this effect was very strong. Younger age at the time of transplantation was previously associated with a faster reconstitution of RTEs, with each additional year of age increasing the time to reach the first RTE percentile by approximately 4% [27]. This age-dependent recovery likely reflects the age-related decline in thymic function, with older patients having less thymic tissue available for T cell production. The importance of age is further emphasized by McAvoy et al., who found that patients over 18 years old had significantly reduced RTE counts compared to younger individuals at 90 days post-HCT [29].

The intensity of conditioning regimens used prior to HCT also plays a significant role in thymic recovery. In our study we had two cohorts of patients with IEI who received similar RIC, RTC and MAC conditioning regimen. In both cohorts the RTE immune reconstitution was slower with increased intensity. In the previous studies this effect was not shown, perhaps due to the heterogeneity of cohorts with malignant and nonmalignant disease transplants [27–29]. The strength and the novelty of our study are the two homogeneous cohorts with the unique patient population with IEI.

The adverse impact of acute GvHD on thymic function and, consequently, on RTE reconstitution is a recurring theme across studies. Similar to results of this study of two independent cohorts, it was previously reported that clinically significant acute GvHD (grades 2 to 4) significantly delayed RTE reconstitution in pediatric HCT recipients [27]. The time to reach age-specific first percentiles for RTEs was 49% longer in patients with severe acute GvHD compared to those without. McAvoy et al. additionally showed that patients with higher grades of acute GvHD experienced delayed RTE recovery, starting as late as 9 to 12 months post-HCT [29]. These observations suggest that the inflammatory environment and potential thymic damage induced by acute GvHD or its treatment hinder the re-establishment of a functional thymic output, leading to prolonged immune deficiency. The association of chronic GvHD with RTE was unable to be evaluated in this study due to small numbers (Cohort A included 0 cases of chronic GvHD).

Additional factors hypothesized to influence RTE unable to be assessed in these cohorts include indication for HCT, use of serotherapy in conditioning, and chronic GvHD. Patients with nonmalignant diseases (NMD) typically exhibit better RTE reconstitution compared to those with malignant conditions. This is likely due to the pre-HCT thymic damage caused by the malignancy itself or by prior treatments such as chemotherapy or radiation. Drozdov et al. observed that patients with nonmalignant diseases had a faster RTE reconstitution compared to those with malignancies, though this effect was less pronounced after adjusting for age and other factors [27]. As the cohorts in this study are uniformly nonmalignant, such comparison is not possible. Further, our small sample size precluded subgroup analysis by type of IEI.

The impact of serotherapy, including dose and timing of administration, on lymphocyte recovery has been well described [35,36]. There is evidence that alemtuzumab affects thymic tissue negatively [37,38]. However, these findings may relate more directly to homeostatic proliferation of mature donor lymphocytes then neothymopoiesis [39]. Unfortunately, we cannot assess the impacts of serotherapy on RTE in this analysis. For Cohort A, serotherapy was uniform across all patients; for Cohort B the type of serotherapy was confounded by conditioning intensity.

We demonstrated a relationship with weak significance between lymphoid chimerism at day 100 and emergence of RTE. However, in the multivariable analysis there was no evidence for this relationship. Unfortunately, we had no measurements of chimerism in isolated RTE cells which would have given more insight into this relationship.

Additionally, we could not correlate RTE values with overall survival due to selection bias of patients who died before one year after HCT, had no RTE measurements and were excluded from the analysis in cohort B.

The findings across these studies have important clinical implications for improving patient outcomes post-HCT. The delayed reconstitution of RTEs in patients with grade 2 to 4 acute GvHD or older age highlights the need for tailored approaches in managing these patients. Strategies to enhance thymic recovery, such as the use of thymopoiesis-promoting agents [40] or GvHD prophylaxis that minimizes thymic damage, could be beneficial. Furthermore, regular monitoring of RTE levels could serve as a valuable tool for assessing thymic function and guiding immunosuppressive therapy to balance the risk of GvHD against the need for effective immune reconstitution [9].

We decided to focus on RTE and not on T-cell receptor excision circles (TRECs) as we believe this method is easier to implement and more generalizable and therefore could be adapted in routine immune monitoring by a majority of transplant centers.

Future research should focus on identifying specific interventions that can accelerate RTE recovery in high-risk populations. This could include exploring the role of novel conditioning regimens that are less toxic to the thymus or investigating the potential of cultured thymic tissue implantation or regenerative therapies to increase thymic function in older patients or those with significant thymic damage. Additionally, larger, multicenter studies are needed to validate the use of RTEs as a predictive biomarker for clinical outcomes including survival and to establish standardized guidelines for their use in posttransplant monitoring.

## CONCLUSION

Serial measurement of RTE is a useful immune reconstitution marker for assessment of thymic function after HCT. In patients with IEI, age at transplantation, occurrence of grade 2 to 4 acute GvHD and intensity of conditioning regimen were strongly associated with slower thymic-derived immune reconstitution.

## Supplementary Material

Supplement

## Figures and Tables

**Table 1:** Baseline Characteristics

		Cohort A	Cohort B
n		34	128
Age at transplantation (median [IQR])		2.15 [0.83, 10.75]	1.41 [0.73, 5.33]
Age group at HCT (%)	< 1 year	12 (35.3)	50 (39.1)
	1 to 7 years	12 (35.3)	56 (43.8)
	>/= 7 years	10 (29.4)	22 (17.2)
Days to neutrophil engraftment (median [IQR])		12.00 [10.25, 13.75]	12.00 [10.00, 13.00]
Sex (%)	Male	23 (67.7)	87 (68.0)
	Female	11 (22.4)	41 (32.0)
IEI diagnosis (%)	Chronic granulomatous disease	7 (20.6)	13 (10.2)
	CD40 ligand deficiency	4 (11.8)	5 (3.9)
	Hemophagocytic lymphohistiocytosis	2 (5.9)	46 (36.2)
	Severe combined immunodeficiency	4 (11.8)	15 (11.8)
	Severe congenital neutropenia	5 (14.7)	2 (1.6)
	Wiskott-Aldrich syndrome	2 (5.9)	16 (12.6)
	Other	10 (29.4)	30 (23.6)
Conditioning intensity(%)	RIC	8 (23.5)	76 (59.4)
	RTC	23 (67.7)	38 (29.7)
	MAC	3 (8.8)	14 (10.9)
GvHD prophylaxis (%)	CNI/MMF	30 (88.2)	30 (23.4)
	CNI/Prednisone		67 (52.3)
	Other	4 (11.8)	16 (12.5)
	T-cell depleted		15 (11.7)
Acute GvHD (%)	No acute GvHD	30 (88.2)	96 (75.0)
	Grade 1	2 (5.9)	11 (8.6)
	Grade 2	2 (5.9)	8 (6.2)
	Grade 3		12 (9.4)
	Grade 4		1 (0.8)
Donor type (%)	MRD	10 (29.4)	24 (18.8)
	MUD	20 (58.8)	93 (72.7)
	UCB	4 (11.8)	4 (3.1)
	MMRD		7 (5.5)

IEI, inborn error of immunity; RIC, reduced intensity conditioning; RTC, reduced toxicity myeloablative conditioning; MAC, myeloablative conditioning; GvHD, graft-versus-host disease; CNI, calcineurin inhibitor; MMF, mycophenolate mofetil; MRD, matched related donor; MUD, matched unrelated donor; UCB, umbilical cord blood; MMRD, mismatched related donor.

**Table 2:** Cohort A Linear Mixed Effects Model

Variable	Estimated Mean Difference in Square-Root of Absolute RTE (95% CI) *P* Values		
	Day 100	6 Months	1 Year
Model 1			
Age	−0.29 (−0.64, 0.06) .10	−0.48 (−0.84, −0.13) .008**	−0.78 (−1.14, −0.42) <.0001****
Conditioning regimen			
MAC versus RIC	−1.70 (−11.25, 7.85) .72	−6.66 (−12.15, 0.89) .090	−9.07 (−19.62, 1.48) .09
MAC versus RTC	−3.27 (−12.18, 5.64) .46	−2.67 (−11.57, 6.22) .55	1.50 (−8.50, 11.50) .76
RIC versus RTC	−1.57 (−7.58, 4.44) .60	3.99 (−2.02, 9.99) .19	10.57 (4.46, 16.68) .001**
aGvHD, grade 2–4 versus 0–1	0.48 (−10.06, 11.02) .93	−0.99 (−11.54, 9.57) .85	−4.47 (−15.11, 6.18) .40
Model 2			
Age	−0.36 (−0.75, 0.04) .08	−0.53 (−0.93, −0.13) .010*	−0.91 (−1.34, −0.48) .0001**
Conditioning regimen			
MAC versus RIC	−2.93 (−13.06, 7.21) .56	−7.31 (−17.44, 2.82) .15	−9.73 (−21.59, 2.12) .10
MAC versus RTC	−3.39 (−12.46, 5.69) .46	−2.76 (−11.83, 6.30) .54	1.20 (−9.20, 11.59) .82
RIC versus RTC	−0.46 (−7.14, 6.22) .89	4.55 (−2.14, 11.23) .18	10.93 (3.98, 17.88) .003**
aGvHD, grade 2–4 versus 0–1	1.18 (−14.14, 16.49) .88	−1.71 (−17.02, 13.61) .82	−11.91 (−27.39, 3.58) .13
Lymphoid donor chimerism, 100 days, full versus mixed	−2.61 (−8.87, 3.65) .41	−1.32 (−7.56, 4.92) .67	−1.22 (−8.36, 5.93) .73

The bold values and the asterics both reflect that these p-values are “significant” -

* P < .05

** P < .01

*** P < .001

**** p < .0001 additionally to the numerical level of significance.

**Table 3:** Cohort B Linear Mixed Effects Model

Variable	Estimated Mean Difference in Square-Root of Absolute RTE (95% CI) *P* Values		
	Day 100	6 Months	1 Year
Model 1			
Age	−0.58 (−1.32, 0.16) .12	−0.60 (−0.93, −0.27) **<.001**	−0.82 (−1.18, −0.46) <.0001****
Conditioning regimen			
MAC versus RIC	−0.82 (−8.48, 6.84) .83	−5.63 (−12.15, 0.89) .090	−13.87 (−19.69, −8.04) <.0001****
MAC versus RTC	−0.33 (−7.64, 6.97) .93	−6.91 (−13.63, −0.18) .044*	−10.01 (−16.12, −3.89) .002**
RIC versus RTC	0.48 (−4.01, 4.98) .83	−1.27 (−5.01, 2.46) .50	3.86 (0.42, 7.29) .028*
aGvHD, grade 2–4 versus 0–1	−1.34 (−7.58, 4.89) .67	−8.48 (−13.61, −3.35) .001**	−7.29 (−11.59, −2.99) .001**
Model 2			
Age	−1.01 (−2.40, 0.38) .15	−0.52 (−0.99, −0.05) .030*	−0.75 (−1.23, −0.28) .003**
Conditioning regimen			
MAC versus RIC	1.56 (−8.63, 11.76) .76	−5.13 (−12.72, 2.47) .18	−15.44 (−22.24, −8.64) <.0001****
MAC versus RTC	1.00 (−9.59, 11.59) .85	−6.21 (−14.68, 2.26) .15	−12.41 (−20.38, −4.44) .003**
RIC versus RTC	−0.56 (−6.86, 5.74) .86	−1.08 (−6.99, 4.82) .71	3.02 (−2.37, 8.42) .26
aGvHD, grade 2–4 versus 0–1	−2.31 (−9.89, 5.28) .54	−8.44 (−15.60, −1.27) .022*	−7.58 (−13.44, −1.72) .013*
Lymphoid donor chimerism, 100 days, full versus mixed	−4.61 (−10.89, 1.67) .15	−4.75 (−10.73, 1.22) .12	−1.20 (−6.66, 4.25) .66

RTE, recent thymic emigrants; MAC, myeloablative conditioning; RIC, reduced intensity conditioning; RTC, reduced toxicity myeloablative conditioning; aGvHD, acute graft-versus-host disease.

The bold values and the asterics both reflect that these p-values are “significant” -

* P < .05

** P < .01

*** P < .001

**** P < .0001 additionally to the numerical level of significance.

## Abbreviations:

CGD: Chronic Granulomatous Disease
HCT: Allogeneic hematopoietic cell transplantation
HLH: Hemophagocytic Lymphohistiocytosis
GvHD: Graft-versus-host disease
IEI: Inborn errors of immunity
IQR: Interquartile range
MAC: Myeloablative conditioning
MMUD: Mismatched unrelated donor
MRD: Matched related donor
MMRD: Mismatched related donor
MUD: Matched unrelated donor
RIC: Reduced intensity conditioning
RTC: Reduced toxicity myeloablative conditioning
RTE: Recent thymic emigrants
