# Supplementary material for: Relevance of Recent Thymic Emigrants Following Allogeneic Hematopoietic Cell Transplantation for Pediatric Patients with Inborn Errors of Immunity
Source: Transplant Cell Ther. Author manuscript; Available in PMC 2025 Apr 1. (PMC11957927; doi:10.1016/j.jtct.2025.02.003)
Supplement: Supplement [file NIHMS2063396-supplement-Supplement.docx]

**Supplementary Material**

**Supplementary Table 1**

**Univariate analyses of RTE associations (Wilcoxon rank sum test p-values)**

|  | **Cohort A** | | | **Cohort B** | | |
| --- | --- | --- | --- | --- | --- | --- |
| **Variable** | **100 days** | **6 months** | **1 year** | **100 days** | **6 months** | **1 year** |
| **Age group at HCT**  **1-7 yrs vs. <1 yr**  **>/=7 yrs vs. <1 yr**  **>/=7 yrs vs. 1-7 yrs** | 0.56  **0.004****  **0.01*** | 0.06  **0.009****  **0.02*** | **0.020***  **0.008****  0.34 | 0.10  **0.02***  0.08 | **<0.01****  **<0.001*****  **<0.01**** | 0.09  **<0.001*****  0.054 |
| **Conditioning regimen**  **MAC vs. RIC**  **MAC vs. RTC**  **RTC vs. RIC** | 0.77  0.96  0.71 | 0.49  0.56  0.06 | 0.39  0.74  **0.03*** | 0.40  0.39  0.81 | **0.03***  0.06  0.46 | **<0.001*****  **<0.01****  0.31 |
| **No or Grade 1 aGVHD vs.**  **Grade 2-4 aGVHD** | 0.78 | 0.53 | 0.22 | **<0.01**** | **<0.001***** | **<0.01**** |
| **Full vs. mixed lymphoid donor chimerism, 100 days** | 0.35 | 0.09 | 0.15 | 0.22 | **0.02*** | 0.0501 |

*p < 0.05, **p < 0.01, ***p < 0.001
